# Flood Routing Model Coupled with Dynamic Leakage Losses for Ephemeral Rivers with Large Potholes

**DOI:** 10.3390/ijerph19137638

**Published:** 2022-06-22

**Authors:** Congmin Liu, Chengzhong Pan, Chunlei Liu, Yuanzheng Zhai, Wanlai Xue, Yongsheng Cui

**Affiliations:** 1 College of Water Sciences, Beijing Normal University, No. 19, Xinjiekouwai St., Haidian District, Beijing 100875, China; 202031470012@mail.bnu.edu.cn (C.L.); chunleiliu0520@163.com (C.L.); diszyz@163.com (Y.Z.); 201931470015@mail.bnu.edu.cn (Y.C.); 2 Beijing Water Science and Technology Institute, No. 21 Chegongzhuang West Road, Haidian District, Beijing 100048, China; xuewanlai@126.com

**Keywords:** ephemeral river, flood routing model, leakage losses, potholes, unsteady flow

## Abstract

Ephemeral rivers commonly occur in regions with a shortage of water resources, and their channel configuration tends to change substantially owing to long drying times and artificial sand extraction. During short-term water conveyance, water storage in large potholes and leakage along the dry riverbed retards the flow, which is detrimental for the river landscape and ecological water demand. The objective of this study is to evaluate the flow process corresponding to a certain release scheme. A coupled dynamic leakage loss and flood routing model was established to predict the flood routing distance for dry rivers with potholes and strong leakage. The model mainly includes three sub-models of flow dynamics, dynamic leakage loss and water balance along multiple cross sections of the river channel. The water head was dominated by flow velocity and the overflow from potholes. The model was applied to Yongding River, a typical ephemeral river in northern China, and the model parameters were calibrated and verified using monitoring data from ecological water releases into the Yongding River in 2019 and 2020, thus, making the model more stable and reliable. Finally, the model was used to evaluate the impact of cross section optimization and pothole treatment on the flow process. This study can provide scientific guidance for ecological water conveyance and the ecological restoration of ephemeral rivers.

## 1. Introduction

Ephemeral rivers, also known as seasonal rivers or non-perennial rivers, refer to those rivers where the river channel is cut off and the riverbed is exposed in the dry season and where the river channel flows or even floods surge in the wet season. Ephemeral rivers constitute more than half of the length of the global river network and will continue to increase in number and length in response to climate change, land-use alteration and water abstraction [1,2,3,4]. Such rivers generally have sandy riverbeds with a high permeability [5,6] as well as uneven permeability along the river channels owing to the size difference in deposited sediment particles [7].

In such dry ephemeral alluvial channels, peak discharge and flood volumes are often reduced downstream and infiltrate into local and regional aquifers, which is the main recharge mode of groundwater in arid and semi-arid areas [8,9]. Tooth (2000) summarized the study on transmission loss in arid rivers of Australia, India, Saudi Arabia and Arizona and reported that the downstream flood volume and peak discharge decreased by 8–95% [10]. Sand excavation events in the dry period led to many large potholes that are distributed along the ephemeral river channel, which seriously change the process of flood routing. These rivers are usually dry, and the ecological environment is degraded; thus, flood routing events are urgently needed [11].

In the context of water shortage, the current strategy is to transform the geometry of the river channel to increase the distance of flood routing and meet the longitudinal connectivity of the river, which is beneficial to the river ecology, riparian environment and urban landscape [12,13]. Therefore, it is crucial to predict the flood routing process under different scenarios, particularly the routing distance.

The most widely used software programs to study the interactions between river and aquifer include MODFLOW [14,15] and HYDRUS [16,17]; however, these software programs focus on groundwater migration rather than surface flood routing. Flood routing calculation models generally adopt the Muskingum [13,18,19] and Saint-Venant equation [20,21] or its simplified form [22,23,24,25]. They are often coupled with the infiltration models to explore the flood routing process with leakage, such as the Green–Ampt model, Philip model, Kostiakov’s empirical formula and Horton equation [26,27].

Cheng and Wang et al., (2015) constructed a flood routing model incorporating intensive streambed infiltration by using the Muskingum–Cunge method combined with the Horton equation. The results of model application showed that the accuracy of the model simulation was high, and the infiltration simulation method could also represent infiltration processes well [28]. However, the number and duration of water conveyance events in ephemeral rivers are limited, coupled with inaccurate measurements owing to the complexity of river sections and scarce gauge record availability in such arid and hyperarid channels [9,29].

As a result, the Muskingum method based on historical hydrological data is no longer applicable. Assumed that the infiltration rate of the river channel was a uniform constant parameter, Morin et al., (2009) added this parameter to the kinematic wave equations to build a flood routing model to simulate the water flow process of Kuiseb River [9]. Ghobadian and Khalaj added Muskat’s equation in Saint-Venant equations to predict the flood routing, and the results showed that the developed model could predict the output hydrograph with high accuracy in consideration of the leakage loss compared with the field data [20].

Due to the complexity of the solution, most of the existing studies simplify the Saint-Venant equation. In addition, the complexity of flood routing on a dry riverbed is higher, and the calculation often needs to give the linear relationship between the discharge and water level on the upper boundary, which is not easy to give. Additionally, the assumptions given in the existing research cannot be proven practical.

To spread downstream in a dry riverbed with substantial leakage, the water must first fill potholes and penetrate into the riverbed [30,31]. Riverbed leakage can seriously affect flood routing [30,32] and even the surface hydrological connectivity between upstream and downstream reaches of dryland rivers occurs only if the runoff propagated into channels overcomes the leakage losses [33,34]. The infiltration rate is uneven along the longitudinal riverbed [35,36,37], and it is very high at first and will gradually decrease with time and eventually tend to be stable [32,38,39].

A constant permeability is commonly used to consider the transmission losses in the river channel [8,9,40,41], which can be assumed as the first approximation only. It is necessary to improve the model according to the temporal and spatial differences of infiltration rate [8,16,42,43]. In addition, the flow process in the potholes differs from that in straight reaches, and water will continue to flow downstream when the pool is full, which seriously affects the flood routing [13].

In this study, we focused on the problems of large potholes, transmission losses and insufficient initial flow in seasonal rivers, divided the channel into multiple reaches on the basis of field investigation, identified the respective riverbed infiltration process, adopted appropriate methods to describe the flood routing process according to the characteristics of the river and established a simplified and coupled dynamic leakage loss and flood routing model. When the discharge process of the upper boundary section was known, the model could calculate the routing of the flow as well as the output parameters, such as the travel distance, arrival time and leakage of the river section.

The model was applied to the southern plain reach of the Yongding River, and the results showed that the model was reliable, which can provide guidelines for the ecological flow processes and channel reconstruction of the Yongding River. This model can also be applied to other similar rivers and has guiding significance for ecological drainage, river ecological restoration and river geometric shape restoration.

## 2. Methods

### 2.1. Model Framework

To simulate the flood routing of ephemeral rivers with large potholes and strong leakage, it was necessary to divide the river channel into different sections, including straight sections with different infiltration characteristics and pothole sections, to calculate their respective flood routing processes. In pothole sections, the water flow almost no longer advances and flows to the next reach only after the pothole is filled with water. Therefore, the water balance method was used to calculate the flow process. In straight sections, a simplified Saint-Venant equation was used to simulate the flood routing.

**Figure 1:**
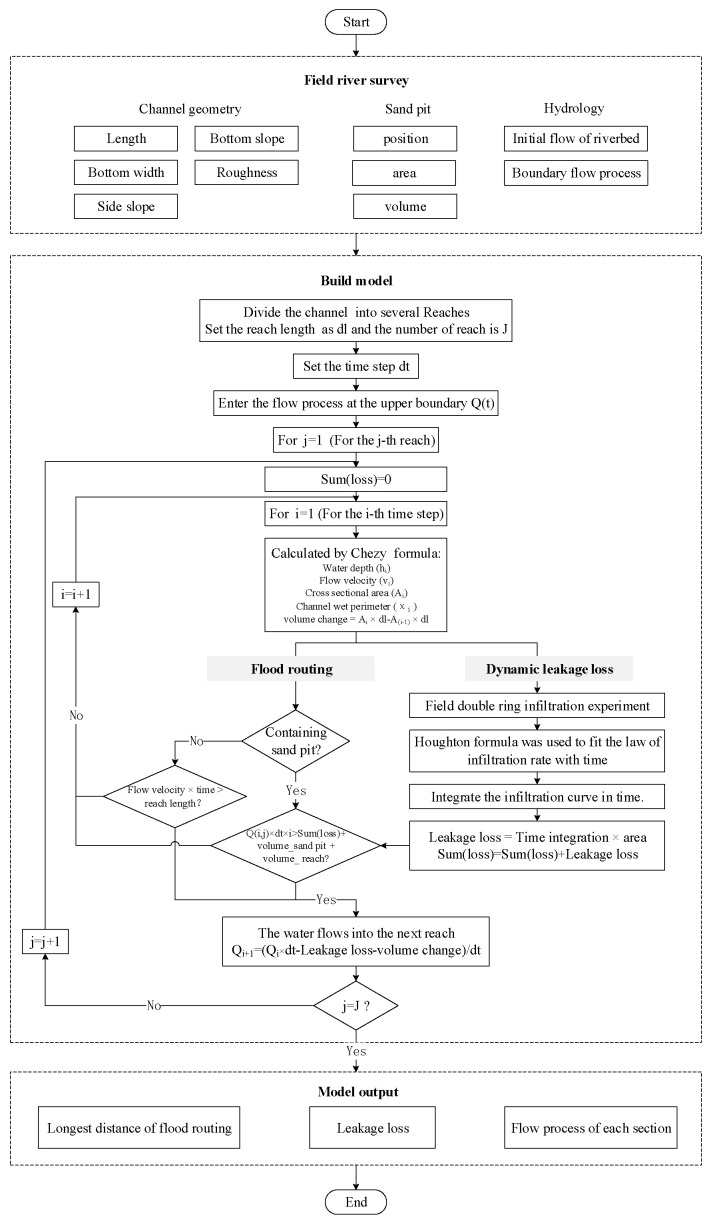
Model framework.

The dynamic leakage losses varies spatially and temporally during the water head advance. The characteristics of riverbed soil will affect the infiltration process; therefore, based on the field experiment, this study fitted the infiltration process using the Horton formula. Owing to the uneven distribution of riverbed lithology along the river, the infiltration process of each river section was explored. In the model, the process of water flow was calculated in turn by appropriately setting the time step, and the infiltration curve was integrated at each time step. This was coupled in the flood routing model in the form of leakage losses. The framework of the model is shown in Figure 1.

### 2.2. Flood Routing

According to the incoming flow and section shape, the hydraulic parameters of each reach were inversely calculated using the Chezy formula. Correspondingly, the parameters, including the water depth, wet perimeter, velocity, cross-section area, hydraulic radius, and dynamic leakage loss of the river reach, can be deduced and used to calculate the inflow process of the next river segment. The model set the length of each reach as L and the time step as dt.

The Saint-Venant equation is generally used for flood routing in open channels, which consists of continuity equation and momentum equation.

Continuity equation:

(1) $$\frac{\partial A}{\partial t}+\frac{\partial Q}{\partial x}=0$$

Momentum equation:

(2) $$\frac{\partial Q}{\partial t}+\frac{\partial \left(Qu\right)}{\partial x}+gA\frac{\partial h}{\partial x}-gAS_{0}+gAS_{f}=0$$

where *A* is the cross-sectional area; *Q* is the flow through the section; *u* is the section velocity; *g* is gravitational acceleration; *h* is the water depth; $S_{0}$ is the river bottom gradient; and $S_{f}$ is the friction gradient,$\;S_{f}=\frac{n^{2}Q\left|Q\right|}{A^{2}R^{\frac{4}{3}}}$.

Equation (1) shows that the change rate of storage is equal to the change rate of flow along the way. Equation (2) shows that the combined action of gravity and pressure allow the water flow to overcome the energy loss caused by inertial force and friction to obtain acceleration. Considering the complexity and limitations of solving Saint-Venant equation, this study simplifies Saint-Venant equation without considering the inertia term and pressure term for the rivers with wide and shallow channel, small slope and slow flow velocity on the plain landform, so that it can be applied to the segmented calculation of such rivers. In the straight sections, it was assumed that the water flow was uniform. According to the inflow, the water depth was inversely calculated using the Chezy formula [44], and then the other hydraulic parameters were obtained. Model parameters including the bottom slope, bottom width and bank slope were obtained from field investigations.

Chezy formula:

(3) $$Q=AC\sqrt{Ri}$$

where *A* is the cross-sectional area; *i* is the bottom slope; *R* is the hydraulic radius, $R=\frac{A}{\chi }$; *χ* refers to the wet perimeter of the cross section; *C* is the Chezy coefficient, $C=\frac{1}{n}R^{\frac{1}{6}}$; *n* refers to the Manning’s roughness coefficient, which was estimated from field observations.

In the straight reaches, the water depth and velocity of the reach were calculated according to the inflow, and then the flow distance advancing with time was calculated at each time step, which was compared with the length of the reach. When the flow distance is greater than the length of the river reach, it indicates that the water flows into the next reach at this time. So there are:

(4) $$l_{j}=\overset{n}{\underset{i=1}{\sum }}V_{j}^{i}\times dt$$

where $V_{j}^{i}$ refers to the velocity of the *j*-th reach at the *i*-th time step. $l_{j}^{i}$ refers to the routing distance of water flow in the *j*-th reach at the *i*-th time step. *i* refers to the number of time steps, *i* = 1, 2, 3⋯. For each time step, the distance $l_{j}$ of the flow was required, when $l_{j}$ > *L*, it indicates that the water flows into the next reach in the *n*-th time step.

In the pothole reaches, the water balance method was used to calculate the time for the pothole to be full of water and predict the outflow process of the pothole. In our study, the proportion of water surface evaporation was less than that of river leakage, so it was ignored. In the water balance method, the income item was the amount of water flowing into the reach from upstream, and the expenditure items were the amount of water flowing out of the downstream section and the amount of water lost through riverbed leakage. The difference between the inflow and outflow was the change of water storage in the reach.

(5) $$W_{j+1}^{i}=\overset{n}{\underset{i=1}{\sum }}W_{j}^{i}-\overset{n}{\underset{i=0}{\sum }}W_{jf}^{i}-V_{j}^{i}$$

where $W_{j}^{i}$ refers to the inflow of the *j*-th reach at the *i*-th time step, m^3^; $V_{j}^{i}$ refers to the water storage of the *j* reach at the *i*-th time step, m^3^; *n* means that the reach is full and begins to produce flow in the *n*-th time step, that is, the flow begins to flow into the next reach.

Whether it was a straight reach or a pothole reach, when there was inflow in the (*j* + 1) reach, the flow was:

(6) $$Q_{j+1}=\frac{Q_{j}^{n}\times dt-W_{j_{f}}^{n}}{dt}$$

where $Q_{j}^{n}$ is the inflow of the *j*-th reach in *n*-th time step, m^3^/s; $Q_{j+1}$ is the inflow of the (*j* + 1)-th reach, m^3^/s.

The upper boundary condition of the model was the flow process of the upstream section. The lower boundary condition of the model was each hydraulic element change with the flow of the incoming water. The initial conditions of the model were that the channel was dry, and the water depth and flow were zero.

### 2.3. Leakage Losses

There is a transition from initial capillary infiltration to later steady-state infiltration [16,39]. During the first phrase of infiltration, the dry sandy soil and the strong capillary pressure gradient during the initial propagation of the wetting front into the soil make infiltration rate of the streambeds extremely high [45], this rate will gradually stabilize with time. In addition, the spatial variability in riverbed lithology along the channel results in great differences in permeability. These initially high, unstable infiltration rates and the heterogeneity of the riverbed make it challenging to estimate leakage using a variety of methods [46]. In this study, we explored the infiltration process of the riverbed in different reaches and integrated the infiltration curve in different time steps, which effectively solved the two problems.

We performed a series of field investigations to examine the infiltration processes. Based on the experimental data, the Horton infiltration model with better fitting effect was selected to fit the infiltration process. It should be noted that other infiltration models may also get better fitting results under different conditions, such as the Green–Ampt model, Philip model and Kostiakov’s empirical formula.

Horton infiltration equation [47,48]:

(7) $$f=f_{c}+\left(f_{0}-f_{c}\right)e^{-kt}$$

where *f* is the infiltration rate; *f_c_* is the stable infiltration rate; *f*_0_ is the initial infiltration rate; *t* is the time; and *k* is the empirical constant related to soil properties.

The accuracy of fitting is evaluated by Residual Error $\delta =\underset{i}{\sum }{\left(Fun\left(t_{i}\right)-Y_{i}\right)}^{2}$, that is, the sum of squares of fitting residuals at each measured point, where $t_{i}$ is the time of the *i*-th monitoring point, $Y_{i}$ is the measured value of the *i*-th monitoring point, and $Fun\left(t_{i}\right)$ is the fitting value at time $t_{i}$.

Owing to the temporal and spatial variation in the dynamic leakage loss, the leakage rate changed dynamically. Therefore, the whole river channel was separated into different reaches to calculate the leakage loss of each reach at each time step. The river segments with potholes and the straight reaches correspond to different calculations of leakage loss.

In the straight reaches:

(8) $$W_{jf}=\overset{N}{\underset{i=0}{\sum }}\chi^{i}\times L^{i}\times \int_{t_{i}}^{t_{i+1}}fd_{t}$$

where $W_{jf}$ refers to the leakage loss in the *j*-th reach; χ refers to the wet perimeter of the reach; *L* is the length of the reach; *f* is the function of the infiltration rate with time; *N* is the number of time steps of water seepage in the *j*-th reach.

In pothole reaches:

(9) $$W_{jf}=Area_{j}\times \overset{N}{\underset{i=0}{\sum }}\int_{t_{i}}^{t_{i+1}}fd_{t}$$

where $W_{jf}$ refers to the leakage loss in the *j*-th reach; $Area_{j}$ refers to the floor area of the pothole in the *j*-th reach.

## 3. Case Study of the Yongding River

### 3.1. Study Area

**Figure 2:**
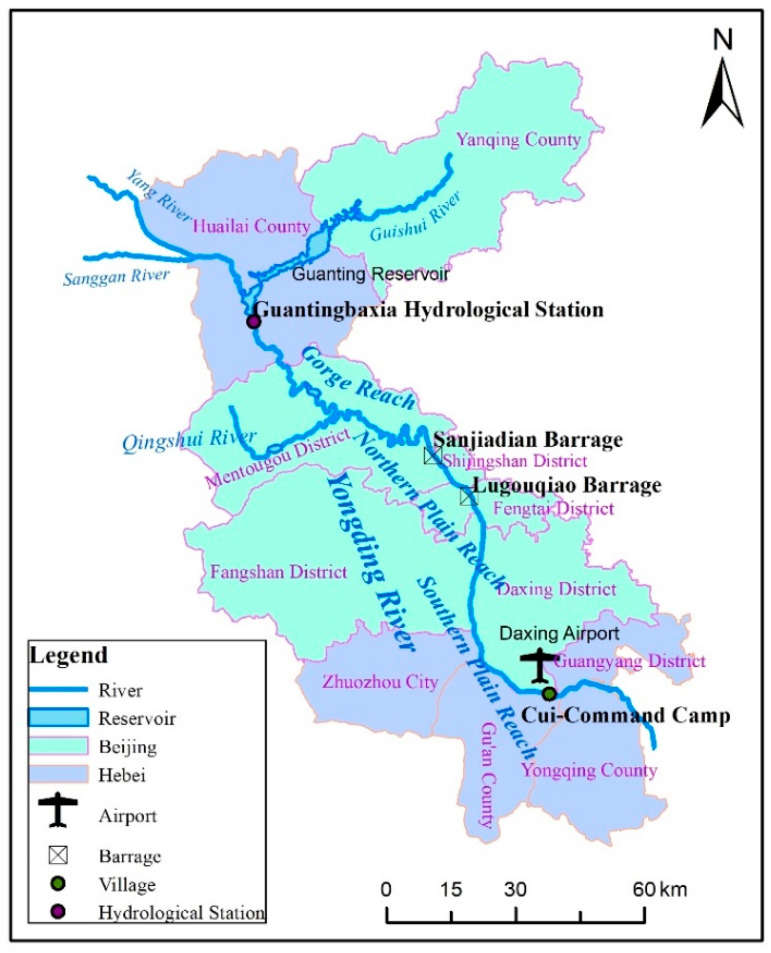
Location and distribution of Yongding River.

The Yongding River originates from the southern edge of Inner Mongolia Plateau and the northern part of Shanxi Plateau and the terrain slopes from northwest to southeast. The Beijing section from Guanting reservoir to the Village of Cui-Command Camp was divided into the Guanting gorge reach (92 km), northern plain reach (37 km) and southern plain reach (64 km), as shown in Figure 2. This study focuses on the southern plain section, which extends from Lugouqiao Barrage to Cui-Command Camp, the water flow in this reach is relatively gentle, with a large amount of sediment deposited, forming a vast alluvial proluvial fan.

**Figure 3:**
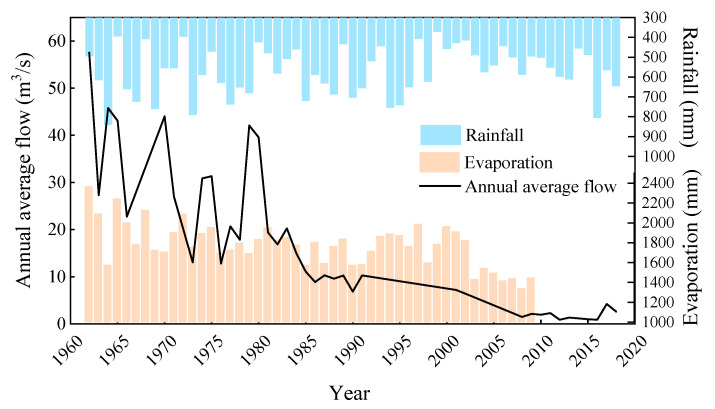
Change process of water resources of Yongding River from 1962 to 2018.

Yongding River is the main river in Beijing. From 1962 to 2018, the average annual rainfall and evaporation [49] of the river reach were relatively stable, and the rainfall was far less than the evaporation. The discharge of Guanting Reservoir in the upstream of the river decreased sharply, as shown in Figure 3, resulting in the cut off and drying up of Yongding River, as shown in Table 1. From 2019 to 2021, Yongding River realized short-term hydraulic connection through artificial water replenishment every year and shown ephemeral characteristics.

Under the condition of insufficient water resources, one of the important management plan of the Yongding River is to promote the flow travels far enough downstream and bypasses Daxing International Airport by modifying the geometry of the river channel, so as to improve the urban landscape of Beijing and gradually repair the ecological environment of the riparian zone.

### 3.2. River Channel

This model focused on the southern plain reach of the Yongding River, from Lugouqiao Barrage to Cui-Command Camp, with a total length of about 64 km. The width of the river channel changed from 400 to 2000 m, there were many large potholes, and the channel was relatively straight, with a sinuosity of less than 1.3 (1.5–2.5 for an average river). In the model, the river channel was divided into 64 reaches, each of which was 1 km long.

According to the field measurements and investigation, the average slope of riverbed i of the first 3 km river segment was approximately 0.005 and that of the 3–64 km reach was approximately 0.00038. As the riverbed is a natural channel on a plain covered by many weeds, grass, woods and potholes, the comprehensive Manning roughness n was estimated according to the measurement data of the experimental flow. The channel was generalized as a wide and shallow trapezoidal channel. Since the flow cannot fill the whole river channel, the bottom width b of the river channel was taken as 50 m on average, according to the actual inundation capacity during historical flow, and the slope m was 5:1.

The channel in the southern plain reach was a wide and shallow sandy channel, and the riverbed has been dry for a long time. In addition, there is a “funnel” phenomenon of groundwater in North China; the surface water of the Yongding River is disconnected from the deeply buried groundwater, resulting in serious leakage in the process of short-term water transmission. As the dynamic leakage loss of the river in the process of water conveyance is large, it has a substantial impact on the water conveyance efficiency of the river, and the water flow is slow to advance.

### 3.3. Potholes

**Figure 4:**
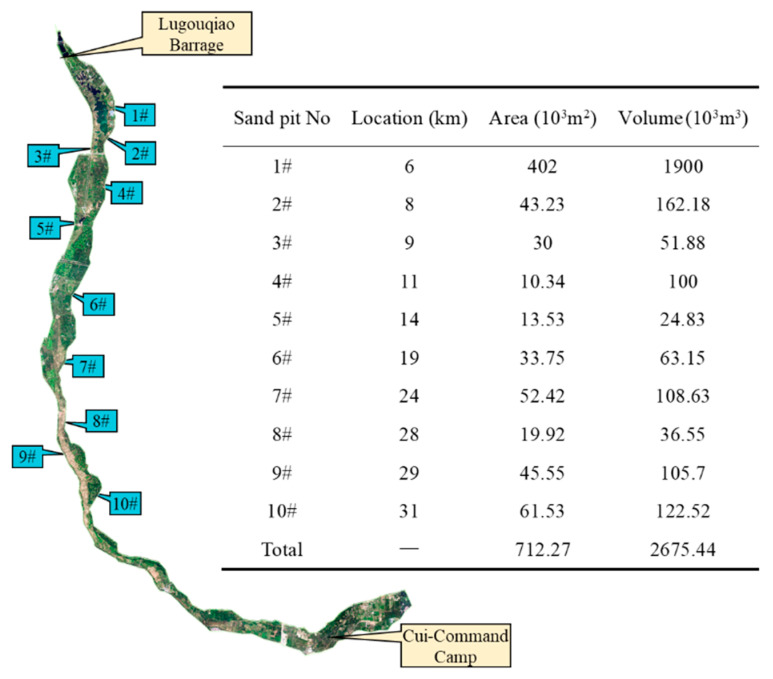
Information of large potholes in southern plain reach.

There were 10 large potholes in the southern plain reach, which were formed by manual excavation during the dry period of the riverbed. The total storage capacity of the 10 potholes is 25.02 × 10^6^ m^3^, which has a substantial impact on the flow routing and cannot be ignored in simulation. The location, area and volume of each pothole are shown in Figure 4.

## 4. Results

### 4.1. Leakage Loss Simulation

The infiltration rate depends on the lithology between the aquifer and the riverbed. The lithology distribution along the river was also highly variable, so the infiltration process of different river sections was very different. The permeability of potholes in the river channel was less than that of the riverbed, and they often stored water for a long time after water delivery in the river channel.

**Figure 5:**
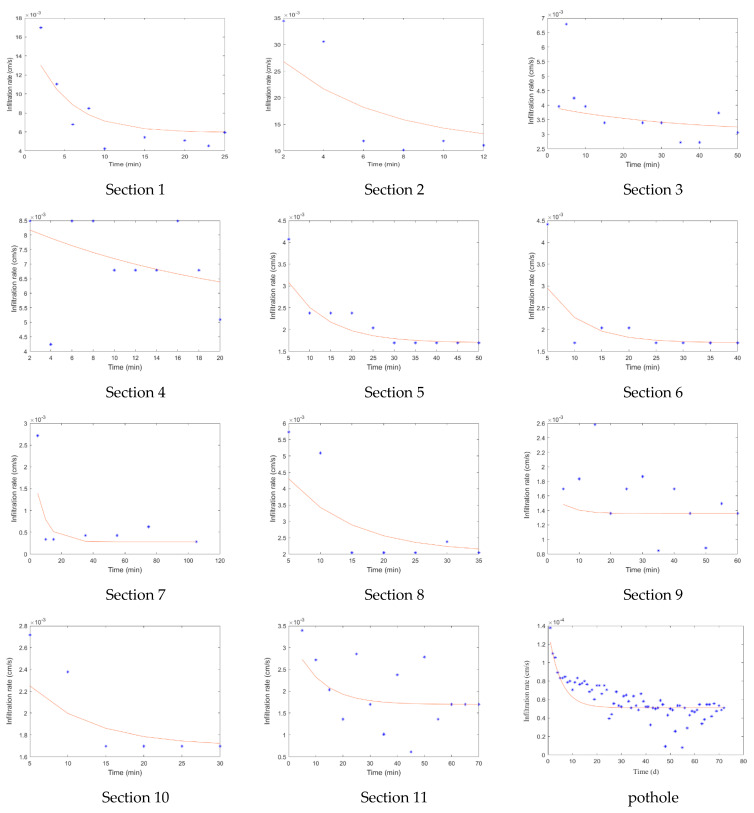
Fitted curve of the infiltration rate of 11 sections and pothole. The blue asterisk indicates the field measured infiltration rate, and the red line indicates the fitted riverbed infiltration process.

The degree of aridity of a dryland river may be an indicator of potential model uncertainty and subsequent attainable predictability of the system. Since the Yongding River water is replenished every spring from April to May, we chose to conduct the infiltration experiment in early April to obtain the infiltration information under this condition. According to the results of riverbed drilling, literature and field investigation, we selected 11 sections to conduct the double ring infiltration experiment. The relationship between the infiltration rate and time of each section was obtained and the Horton infiltration equation was fitted to each of the 11 sections. In addition, we conducted the same experiment for potholes and obtained their infiltration equation. The relevant data are shown in Table 2, and the fitted figures are shown in Figure 5.

According to Table 2 and Figure 5, the experimental data and fitted results showed that the sediment particle size deposited along the river formed by alluvial proluvial deposits in the plain area gradually decreased, which in turn led to a decreasing trend in the permeability of the riverbed along the river.

### 4.2. Model Calibration

Owing to the shortage of the Yongding River flood events, this study used the flow process in the spring of 2019 to calibrate the model parameters, mainly to modify the bottom slope *i*, roughness coefficient *n* and side slope *m*. In the spring of 2019, Lugouqiao Barrage discharged water downstream for 52 days from 17 April to 8 June. From 17 April to 16 May, the discharge flow was 5–13 m^3^/s. From 17 May, the flow increased, averaging 20–30 m^3^/s. From 9:00 on 5 June to 20:00 on 6 June, the accumulated water in the barrage was used for a large flow discharge, with a flow of 40–60 m^3^/s. The discharge was completed at 13:00 on 8 June, with a total discharge of 59 × 10^6^ m^3^ of water downstream. This was the largest ecological water supplement in more than 20 years.

Lugouqiao Barrage began to lift its gate on 17 April, after 10 days, the water entered the pothole at Jingliang Road on 27 April, and the cumulative discharge from Lugouqiao was 8 × 10^6^ m^3^. On 3 June, the pothole of Jingliang Road was filled, and the water overflowed the pit. The water discharged from Lugouqiao overflowed Jingliang Road pothole for a total of 47 days and amounted to 50 × 10^6^ m^3^. On 9 June, after 53 days, the flow finally reached 800 m under the Huangliang Railway Bridge, 15 km away from Lugouqiao, and the total discharge from Lugouqiao was 59 × 10^6^ m^3^.

**Figure 6:**
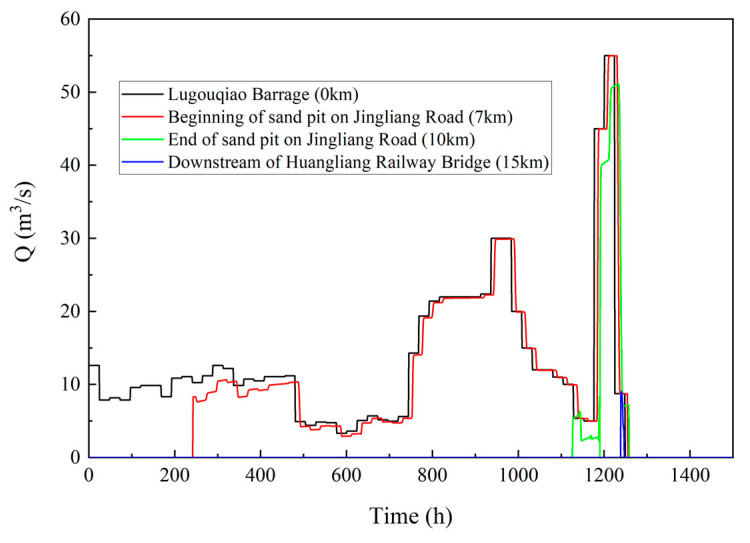
Flow process diagram of typical sections in the southern plain reach of the Yongding River in 2019.

The results of the calibrated model are shown in Table 3. From the three aspects of water flow travel distance, arrival time and water demand, the results were consistent with the measured variables, indicating that the model construction was satisfactory. The model also calculated the leakage losses and the flow process at each section along the way, as shown in Table 3 and Figure 6 below.

The calibration results of the main parameters of the model are shown in Table 4. These calibrated parameters improved the model accuracy in the simulation of the Yongding River. The relative error of this model can be expressed as follows:

(10) $$\varepsilon =\frac{\sqrt{\left(\sum^{\;}{\left(Y_{i}-y_{i}\right)}^{2}\right)}}{\sum^{\;}y_{i}}\times 100\%$$

The relative error in flow routing duration $\varepsilon_{t}$ is 0%, and that in flow discharge $\varepsilon_{q}$ is 1.16%, which indicates that the accuracy of the model is very high.

### 4.3. Model Validation

To more scientifically allocate the water resources downstream in the southern plain reach and realize the goal of connecting the Yongding River to Daxing International Airport, a pulse ecological dispatch scheme was formulated before the ecological water replenishment in the spring of 2020, according to the water amount that can be discharged from the Lugouqiao Barrage. The Lugouqiao gate began to lift and discharge at 8:00 on 28 April and closed at 17:20 on 14 May, lasting 16.4 days with a cumulative discharge of 95 × 10^6^ m^3^.

From 28 April to 5 May, the dispatch mode of “discharge is equal to inflow” was adopted to replenish water to the southern plain reach. From 5 May to 14 May, a pulse test dispatch was conducted. By reducing the discharge of the reservoir in advance and raising the water level upstream of the gate, two pulse discharge tests were performed on 8 and 14 May, lasting 10 and 4.5 h, respectively. The maximum discharge was 317 m^3^/s, which was close to the 3-year return flow.

The flow reached the ethylene pipe bridge 10 km downstream at 13:30 on 3 May (takes 125.5 h), reached the Sixth Ring Road at 7:30 on 5 May (takes 167.5 h) and reached the boundary of Cui-Command Camp at 15:18 on 12 May (takes 343.3 h). The Beijing section of the Yongding River was penetrated by water flow for the first time in 25 years. As of 25 May, Cui-Command Camp had a cumulative discharge of 13.65 × 10^6^ m^3^.

**Figure 7:**
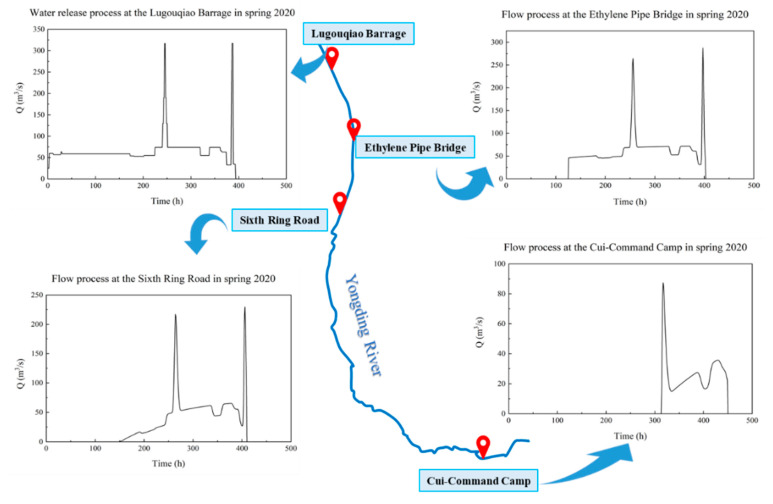
Flow process of typical sections in the southern plain reach of the Yongding River in spring 2020.

The results of the model were consistent with the actual flow process, as shown in Table 5. The discharge process of the Lugouqiao Barrage and the flow process in typical sections are shown in Figure 7. The relative error of this model in flow routing duration $\varepsilon_{t}$ is 0.12%, and that in flow discharge $\varepsilon_{q}$ is 2.78% (Use Formula (10)). In general, the accuracy of the model is very high, which can provide scientific guidance for the upstream water release work.

The leakage from three large potholes with time, the total leakage from ten potholes with time and the leakage from the river channel (excluding potholes) with time during the process of water conveyance are shown in Table 6 below.

Overall, the flow routing speed in the plain section gradually accelerated from north to south. The main factors affecting flow routing speed were channel storage, pothole storage and leakage loss. The pothole 1 and pothole 2 above Jingliang road had the largest storage capacity and the slowest water flow. The Zhuozhou section of Hebei below Jingliang road and Gu’an Jingjiu Railway section had the smallest storage capacity and the fastest water flow.

### 4.4. Model Application for Channel Geometric Reconstruction

The substantial shortage of water resources in seasonal rivers formed by alluvium and the wide cross section of these rivers means that, when limited water resources are released into the dry riverbed, the water flow often stops because of leakage. Considering the medium- and long-term ecological restoration of the Yongding River, the model was able to assume different cross-sectional river shapes. The water flow travel distance under different cross-sectional restoration methods and different pothole series methods as well as the total water volume required to meet the water conveyance of the whole river can be calculated. In this paper, the parameters of river flow were compared and analyzed using the restoration of different bottom widths and pothole landfill restoration as examples, as follows.

#### 4.4.1. Cross Section Optimization

As the cross section of Yongding River is wide, and there is a shortage of water resources from upstream, the water flow only travels through narrow and deep grooves in low terrain. For the medium- and long-term ecological restoration of the Yongding River, manual excavation of the river channel is being considered to repair the cross-section shape. With reference to the actual discharge width in the river channel, the bottom width in the model was set as 10, 20, 30, 40, 50, 60, 80 and 100 m. In the medium and long term, there will still be a shortage of water resources in the southern plain reach of Yongding River. The water flow process in the southern section of the plain was calculated according to the discharge process of the Lugouqiao Barrage in spring 2019. The results are shown in Table 7.

Based on the water resource conditions and ecological water requirements of the southern plain reach of the Yongding River, the current river cross section is too wide, which is not conducive to the advancement of water flow. It is suggested that the cross-section shape can be adjusted in the later stage. According to the model and comparative analysis of the flow process, the bottom width of the southern plain reach of the Yongding River should be 30 m. In the case of water shortage, the water volume of 59 × 10^6^ m^3^ can ensure the flow through the whole channel. The adjusted narrow width is also more conducive to reducing riverbed leakage and allows water flow for a longer distance than does the wide channel.

#### 4.4.2. Potholes Treatment

**Figure 8:**
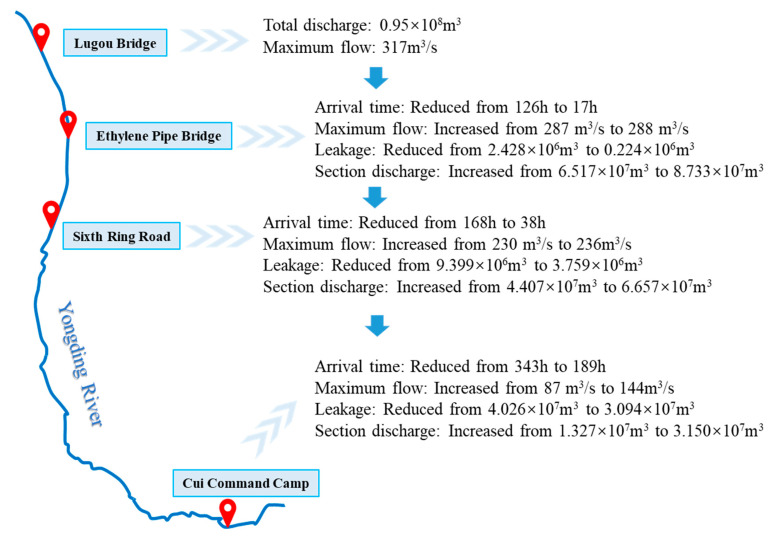
Comparison of water flow routing process before and after pothole repair.

In the spring of 2020, the water flowed downstream through the southern plain reach of the Yongding River; however, there were ten potholes along the way with a substantial water storage capacity (see Table 2), which hindered the flow process. Considering the medium- and long-term planning of ecological restoration of the Yongding River, and assuming that the potholes are repaired, the model was used to simulate the different situations of water passing through and bypassing the potholes. The model simulation scenario was set to release water from the Lugouqiao Barrage based on the spring flows of 2020. A comparison of the water flow routing process and riverbed leakage before and after pothole repair is shown in Figure 8.

After the potholes were repaired, the water reached each section in a shorter time. According to the discharge process of the Lugouqiao Barrage in 2020, the time the water took to reach Cui-Command Camp was reduced from 343 to 189 h, and the whole water flow process advanced by 6.4 d. In addition, owing to the reduction of water storage in the potholes, the transmission flow of the river increased, which changed the hydraulic process of the river and reduced leakage. After the repair of the potholes, the total leakage from Lugouqiao to Cui-Command Camp reduced from 4.026 × 10^7^ m^3^ to 3.094 × 10^7^ m^3^, and the total water demand of the river reduced from 8.099 × 10^7^ m^3^ to 3.951 × 10^7^ m^3^. 

In summary, where there is a shortage of river water, it is recommended that the potholes are filled in or the river channel is excavated to bypass the potholes. This will reduce the water storage capacity of the river channel, help promote the water flow to bypass Daxing International Airport to Cui-Command Camp and realize the goal of water supply for the whole river in the southern plain reach. This is important for repairing the ecological environment of the riparian zone and maintaining the urban river landscape.

## 5. Discussion

Ephemeral rivers are dry for long periods, and there is substantial leakage in the process of short-term water conveyance. To consider the impact of transmission losses on flood routing, the segmented infiltration process of the riverbed was fitted based on the field experiment. Considering the temporal and spatial difference of infiltration, a coupled dynamic leakage loss and flood routing model was constructed. According to the fitted results of the experimental data, the sediment particles deposited along the river formed by alluvial proluvial deposits in the plain area gradually decreased, which led to a trend of decreasing permeability along the riverbed.

Considering the large potholes in the riverbed and insufficient initial flow, a flood routing model in line with the actual flow process of each section was established. The model was based on the basic principles of physics, and the calculation was simple. If ephemeral rivers are dry for long periods, the river ecological environment can become degraded. On the one hand, the ecological water demand of the riparian zone mainly depends on the leakage recharge during river water delivery, and the water delivery distance is directly related to the scope of “irrigation” of the riparian zone through leakage. On the other hand, river ecology requires water flow throughout the river. 

For urban rivers, the water transmission distance is important for the river ecology and the urban ecological environment, particularly for rivers close to urban landmark buildings (for example, Yongding River bypasses Daxing International Airport). Therefore, the flood advance distance is an important parameter of flood routing. Our model is applicable for the calculation of the flow and leakage of similar ephemeral rivers and forms an important reference for the formulation of water-release schemes.

Based on the current situation of the Yongding River water resources and conveyance objectives, the model was applied to calculate the effect of river cross-section restoration and pothole repair. The results showed that a bottom width of 30 m and pothole repair have significant effects on the advancement of water flow. Therefore, this model can provide guidelines for the reasonable medium- and long-term ecological restoration of rivers. When conditions permit, the model would benefit from more flow events for parameter calibration and verification to improve the accuracy of the model.

## 6. Conclusions

The model considered the influence of large potholes, the temporal and spatial difference of riverbed permeability and the lack of initial flow. We found that sediment particles deposited along the river formed by alluvial proluvial deposits in the plain area gradually decreased in size, which also decreased the permeability of the riverbed along the river. Owing to the difference in landforms and deposited sediment size of the riverbed bottom, the river channel was divided into 11 zones and potholes to ascertain the respective infiltration processes. A simplified Saint-Venant equation was coupled with the Horton infiltration equation to construct a model of water flow routing. The model reliably predicted the flow process of the Yongding River.

On the basis of river investigation, the model can be applied to the flood routing of other ephemeral rivers. This coupled model not only predicts the water leakage loss and travel process of the water head but also provides important guidance for river reconstruction and ecological restoration. In addition, this interdisciplinary study linking flood routing, leakage loss (hydrology), infiltration (hydrology/soil physics), riparian ecological environment (ecology) and aquifer recharge (hydrogeology) will greatly contribute to a general understanding of the artificial water transfer process of ephemeral rivers.

## Figures and Tables

**Table 1:** Average drying up statistics for the Yongding River (Beijing section) from 2005 to 2014.

Reach	Initial Section	Termination Section	Length (km)	Dry Days (d)	Cut-Off Days (d)
Gorge section	Guanting Reservoir	Sanjiadian Barrage	92	0	120
North section of plain	Sanjiadian Barrage	Lugouqiao Barrage	37	0	365
South section of plain	Lugouqiao Barrage	Cui-Command Camp	64	360	365

**Table 2:** Fitting parameters of infiltration process in the 11 sections and the potholes.

Section	Location (km)	*f*_0_ (cm/s)	*f_c_* (cm/s)	Coefficient *k*	Horton Equation (cm/s)	Residual Error δ
1	20	0.0170	0.0059	0.2219	*f*_2_ = 0.0059 + (0.0170 − 0.0059) × *e*^−0.2219*t*^	3.30 × 10^−5^
2	25	0.0144	0.0110	0.0356	*f*_3_ = 0.0110 + (0.0144 − 0.0110) × *e*^−0.0356*t*^	2.97 × 10^−4^
3	28.5	0.0040	0.0031	0.0308	*f*_4_ = 0.0031 + (0.0040 − 0.0031) × *e*^−0.0308*t*^	1.10 × 10^−5^
4	33	0.0085	0.0051	0.0482	*f*_5_ = 0.0051 + (0.0085 − 0.0051) × *e*^−0.0482*t*^	2.06 × 10^−5^
5	44	0.0041	0.0017	0.1080	*f*_6_ = 0.0017 + (0.0041 − 0.0017) × *e*^−0.1080*t*^	1.25 × 10^−6^
6	47	0.0044	0.0017	0.1548	*f*_7_ = 0.0017 + (0.0044 − 0.0017) × *e*^−0.1548*t*^	2.53 × 10^−6^
7	48.5	0.0027	0.0003	0.1570	*f*_8_ = 0.0003 + (0.0027 − 0.0003) × *e*^−0.1570*t*^	2.14 × 10^−6^
8	52	0.0037	0.0020	0.0513	*f*_9_ = 0.0020 + (0.0037 − 0.0020) × *e*^−0.0513*t*^	5.60 × 10^−6^
9	53	0.0017	0.0014	0.2000	*f*_10_ = 0.0014 + (0.0017 − 0.0014) × *e*^−0.2000*t*^	2.68 × 10^−6^
10	58.6	0.0027	0.0017	0.1227	*f*_11_ = 0.0017 + (0.0027 − 0.0017) × *e*^−0.1227*t*^	4.00 × 10^−7^
11	59	0.0034	0.0017	0.0995	*f*_12_ = 0.0017 + (0.0034 − 0.0017) × *e*^−0.0995*t*^	5.41 × 10^−6^
potholes		1.3773 × 10^−4^	5.0926 × 10^−5^	0.2000	*f_sk_* = 5.0926 × 10^−5^ + (1.3773 × 10^−4^ − 5.0926 × 10^−5^) × *e*^−0.2*t*^	1.371 × 10^−8^

**Table 3:** The results of the water flow routing process in spring 2019.

Typical Section	Arrival Time	Lugouqiao Discharge	Leakage along the Way
Begin of pothole on Jingliang Road (7 km)	243 h (Day 10)	0.084 × 10^8^ m^3^	0.035 × 10^8^ m^3^
End of pothole on Jingliang Road (10 km)	1127 h (Day 47)	0.487 × 10^8^ m^3^	0.228 × 10^8^ m^3^
Huangliang Railway Bridge (15 km)	1270 h (Day 53)	0.59 × 10^8^ m^3^	0.326 × 10^8^ m^3^

**Table 4:** Calibration of the main parameters of the model.

Parameter	Before Calibration	After Calibration
bottom slope *i* of 0–3 km	0.005	0.005
bottom slope *i* of 3–64 km	0.00038	0.00026
roughness coefficient n	0.07	0.04
side slope m	5:1	5:1

**Table 5:** The results of the water flow routing process in spring 2020.

Typical Section	Arrival Time	Lugouqiao Discharge	Leakage along the Way
Ethylene Pipe Bridge (10 km)	126 h (3 May)	0.265 × 10^8^ m^3^	0.024 × 10^8^ m^3^
Sixth Ring Road (17 km)	168 h (5 May)	0.354 × 10^8^ m^3^	0.094 × 10^8^ m^3^
Cui-Command Camp (64 km)	343 h (12 May)	0.810 × 10^8^ m^3^	0.403 × 10^8^ m^3^
As of 25 May, Cui-Command Camp’s total outbound water volume was 13.27 × 10^6^ m^3^			

**Table 6:** Leakage loss over time.

Time	Travel Distance	Leakage Lose (m^3^)				
		Pothole 1 (6 km)	Pothole 2 (8 km)	Pothole 3 (9 km)	Total Leakage of Ten Potholes	River Course (Excluding Potholes)
10 h	5 km	0	0	0	1.09 × 10^4^	4.69 × 10^4^
40 h	7 km	3.11 × 10^5^	0	0	3.70 × 10^5^	3.78 × 10^5^
70 h	7 km	5.73 × 10^5^	0	0	6.68 × 10^5^	6.75 × 10^5^
100 h	7 km	7.78 × 10^5^	0	0	9.01 × 10^5^	9.34 × 10^5^
126 h	10 km	9.18 × 10^5^	1.77 × 10^4^	4.56 × 10^3^	1.08 × 10^6^	1.34 × 10^6^
130 h	11 km	9.37 × 10^5^	2.24 × 10^4^	8.07 × 10^3^	1.11 × 10^6^	1.72 × 10^6^
160 h	16 km	1.06 × 10^6^	5.30 × 10^4^	3.10 × 10^4^	1.33 × 10^6^	6.34 × 10^6^
168 h	17 km	1.09 × 10^6^	6.00 × 10^4^	3.62 × 10^4^	1.37 × 10^6^	7.98 × 10^6^
190 h	19 km	1.16 × 10^6^	7.69 × 10^4^	4.88 × 10^4^	1.51 × 10^6^	1.23 × 10^7^
220 h	19 km	1.23 × 10^6^	9.55 × 10^4^	6.27 × 10^4^	1.66 × 10^6^	1.71 × 10^7^
250 h	24 km	1.29 × 10^6^	1.10 × 10^5^	7.36 × 10^4^	1.79 × 10^6^	2.18 × 10^7^
280 h	31 km	1.34 × 10^6^	1.21 × 10^5^	8.20 × 10^4^	1.95 × 10^6^	2.67 × 10^7^
310 h	54 km	1.37 × 10^6^	1.30 × 10^5^	8.86 × 10^4^	2.15 × 10^6^	3.21 × 10^7^
343 h	64 km	1.40 × 10^6^	1.36 × 10^5^	9.28 × 10^4^	2.32 × 10^6^	3.79 × 10^7^

**Table 7:** The results of modelled trapezoidal sections under different bottom widths.

Bottom Width	Time to Reach Typical Section (h)			Maximum Distance of Water Flow (km)	Outflow Volume (m^3^)	Leakage along the Way (m^3^)
	Ethylene Pipe Bridge (10 km)	6th Ring Rd (17 km)	Cui-Command Camp (64 km)			
10 m	844	887	1273	64	2.744 × 10^6^	2.505 × 10^7^
20 m	850	903	1286	64	1.475 × 10^6^	2.672 × 10^7^
30 m	854	938	1297	64	0.457 × 10^6^	2.804 × 10^7^
40 m	860	960	-	53 (1294 h)	0	2.844 × 10^7^
50 m	865	978	-	44 (1285 h)	0	2.889 × 10^7^
60 m	871	1080	-	31 (1258 h)	0	2.972 × 10^7^
80 m	882	1200	-	24 (1240 h)	0	3.208 × 10^7^
100 m	892	1201	-	19 (1220 h)	0	3.262 × 10^7^

Note: The numbers in brackets refer to the time taken for the flow to reach the longest distance.

## Data Availability

Not applicable.
